# Uncertainties in linac primary barrier transmission values

**DOI:** 10.1002/acm2.13574

**Published:** 2022-03-02

**Authors:** Patrick N. McDermott, Michael D. Sigler, Ian P. Lake, Danielle Lack

**Affiliations:** ^1^ Beaumont Health System Royal Oak Michigan USA; ^2^ Beaumont Health System Troy Michigan USA

**Keywords:** primary barrier, linac, transmission, TVL

## Abstract

Primary barrier design for linac shielding depends very sensitively on tenth value layer (TVL) data. Inaccuracies can lead to large discrepancies between measured and calculated values of the barrier transmission. Values of the TVL for concrete quoted in several widely used standard references are substantially different than those calculated more recently. The older standard TVL data predict *significantly lower* radiation levels outside primary barriers than the more recently calculated values under some circumstances. The difference increases with increasing barrier thickness and energy, and it can be as large as a factor of 4 for 18 MV and concrete thickness of 200 cm. This may be due to significant differences in the beam spectra between the earlier and the more recent calculations. Measured instantaneous air kerma rates sometimes show large variations for the same energy and thickness. This may be due to confounding factors such as extra material on, or inside the barrier, variable field size at the barrier, density of concrete, and distal distance from the barrier surface. In some cases, the older TVL data significantly underestimate measured instantaneous air kerma rates, by up to a factor of 3, even when confounding factors are taken into account. This could lead to the necessity for expensive remediation. The more recent TVL values tend to overestimate the measured instantaneous dose rates. Reference TVL data should be computed in a manner that is mathematically consistent with their use in the calculation of air kerma rate outside barriers directly from the linac “dose” rate in MU/min.

## 1 INTRODUCTION

This study was prompted by radiation measurements made for primary barriers for a newly constructed vault at one of our clinics (Lenox Twp, MI, USA). The primary barriers are two concrete side walls and the concrete roof. The linac is an Elekta Versa HD with beam energies of 6, 10, and 15 MV. The measured instantaneous radiation levels for 15 MV (field size 40 cm × 40 cm, collimator angle 45°, 700 MU/min), made with multiple survey meters, were up to a factor of 2.5 times larger than predicted based on the tenth value layer (TVL) data of NCRP Report No. 151 (hereafter NCRP151).^1^ The calculations of the expected instantaneous dose rates (IDR) were checked by two other physicists who found no error. The survey measurements were made by multiple physicists on several occasions using different calibrated ionization chamber survey meters.

The calculation of primary barrier radiation levels is relatively straightforward and it was surprising that measured values were so much higher than predicted. These measurements led to an investigation. Laser mounting was initially suspected. The side lasers are mounted in a recess in the finished wall but there is no recess in the concrete wall. Furthermore, they are mounted on an aluminum plate that should slightly *reduce* the expected IDR.^1^ The thickness of the concrete in the side walls was verified as 72 in. as expected and designed. There is no evidence of voids in the concrete. At 15 MV the thickness would need to be 66 in. for the IDR to match NCRP151 predictions. The density of the concrete was certified as 142.9 lb/ft^3^, this is equivalent to 2.29 g/cm^3^. The density assumed in NCRP151 is 2.35 g/cm^3^. This implies that our 72 in. thick concrete is equivalent to about 70 in. of NCRP concrete. This is too small a difference to explain the discrepancy in readings. Alternatively, the density would have to be about 9% lower than 2.35 g/cm^3^ for the measurements to agree with NCRP151. According to Martin and McGinley^2^ the density of concrete is usually specified wet. The dry density is expected to be ∼2% lower. Further detail can be found in Section 3.

These considerations led to a suspicion that perhaps the barrier transmission values are inaccurate. The barrier transmission is very sensitive to the values of the TVL, as these numbers appear in an exponent. In this study, a comparison is made among various published TVL data for concrete primary barriers and between measured IDR and predicted values. There are differences as large as a factor of 4 in calculated barrier transmission among published TVL data sets. In addition, NCRP151 TVL data can underestimate measured IDR by as much as a factor of 3.

## 2 METHODS

In Section 3, the differences in calculated barrier transmission based on published TVL data between widely quoted references are discussed along with the implications. These references are the NCRP151, IAEA Report No. 47 (hereafter IAEA47^3^), IPEM Report No. 75 (second edition, hereafter IPEM75^4^), and the more recently calculated values by Jaradat and Biggs^5^ (hereafter JB) and Karoui and Kharrati^6^ (hereafter K2).^1^ The methods by which various sets of TVL data have been computed will be compared and contrasted. A comparison is also made between measured and predicted IDR based on the various TVL data. The field size dependence of barrier transmission, *B*, is not discussed in NCRP151, IAEA47, or IPEM75. The large variation in *B*, with field size based on the JB TVL data is discussed below.

## 3 RESULTS

### 3.1 Published TVL data

The values of the TVL for concrete quoted in many standard references (NCRP151, IPEM75, Martin and McGinley) can all be traced back to a 1984 paper by Nelson and LaRiviere^7^ (hereafter NL).^2^ The origin of the IAEA47 TVL data is unclear as there is no reference, but a footnote says: “Adapted from Varian Associates.”^3^ It is to be noted that one of the NL authors was affiliated with Varian.^2^ In the NL paper, values of the first, second, and third concrete 10th value thickness (TVL_1_, TVL_2_, and TVL_3_) have been calculated for beam energies of 6, 10, and 25 MV. It appears as if the values quoted in NCRP151 for 15 and 18 MV are based on interpolations between the NL values at 10 and 25 MV. As stated in NCRP151, TVL values for 4 MV are based on an extrapolation, most likely from the NL values for 6 and 10 MV.

The NL barrier transmission is defined as B=D(x)/D(0), where

(1) $$\begin{array}{c} D\left(x\right)=\frac{1.6\times 10^{-8}}{d^{2}}\int_{E_{\min}}^{E_{0}}E\;S(E){\left(\frac{\mu_{\mathrm{en}}}{\rho }\right)}_{a}B\left(E,\mu x\right)e^{-\mu x}dE \end{array}$$

and *S*(*E*) is the “photon energy spectrum per incident electron” in units of MeV^−1^ sr^−1^ per incident electron, B(E,μx) is the absorbed dose build‐up factor, *x* is the thickness of the barrier, *μ* is the linear attenuation coefficient, and ${\left(\mu_{\mathrm{en}}/\rho \right)}_{a}$ is the mass‐energy absorption coefficient for air.^7^ The numerical factor in front of the integral sign is irrelevant as it divides out. The build‐up factor (Berger form) uses parameters taken from a 1966 publication based on an isotropic source in an infinite medium.^8^ In Equation (1), “D(x)” is actually the air collision kerma and therefore D(0) is not the dose in water in a large water phantom. Thus D(0) is neither easily tied to the rep rate (in MU/min) of the linac nor to the calibrated dose rate in a large water phantom. The NL field size is unclear, but is presumed to be large. It should be emphasized that the NL TVL values were *not* computed using a Monte Carlo algorithm, even though the beam spectra were calculated using the EGS code.

In 2007, in a paper by JB, new values of TVL_1_, TVL_2_, and TVL_3_ for concrete have been computed using the Monte Carlo code MCNP.^5^ These calculations utilized the linac energy spectra computed by Sheikh‐Bagheri and Rogers.^9^ These spectra reproduce measured depth dose curves for Elekta and Varian linacs very accurately. The TVL calculations were performed for energies of: Co‐60, 4, 6, 10, 15, and 18 MV. The TVL have been computed for circular fields with half opening angles of 0°, 3°, 6°, 9°, 12°, and 14°. This is the angle between the central axis and the edge of the circular field. The distance to the distal surface of the barrier was fixed at a nominal value of 6.0 m.^3^ The values of the TVL were computed for distances from the distal surface of the barrier of *d*_w_ = 0.3, 1.0, and 2.0 m. Concrete thickness ranged from 76.5 to 151.5 cm.

Arguments in favor of the improved accuracy of JB TVL values in comparison to NL are: (a) they were computed using a Monte Carlo algorithm; (b) they use more recently computed linac beam spectra; and (c) they do not rely on energy interpolation or extrapolation for 4, 15, and 18 MV. On the other hand, the barrier transmission computed by JB appears to ignore the substantial change in the energy spectrum of the beam as it emerges from the barrier. JB have used the MCNP tally “F4” based on photon fluence only. According to Shultis and Faw, the MCNP F4 tally is given by:

(2) $$F4=\frac{1}{V}\int_{V}dV\int_{E}dE\int_{4\pi }d\Omega \;\;\Phi (\vec{r},E,\Omega ),$$

where *V* is the volume of the cell, *E* is the photon energy, *Ω* is the solid angle, and $\Phi (\vec{r},E,\Omega )$ is the differential energy and angular distribution of the fluence.^10^ F4 is thus the total fluence and not the air kerma. The barrier transmission calculated by JB appears to be simply the ratio of the total fluence with the barrier present to that in the absence of the barrier.

An alternative, and arguably better, definition of the barrier transmission can be defined as the ratio of the air collision kerma with the barrier present to that in the absence of the barrier (as in Equation (1)). This ratio is given by:

(3) $$B_{k}\left(t\right)=\frac{\int_{0}^{\infty }E\;\Phi \left(t,E\right){\left(\mu_{\mathrm{en}}/\rho \right)}_{a}dE}{\int_{0}^{\infty }E\;\Phi \left(0,E\right){\left(\mu_{\mathrm{en}}/\rho \right)}_{a}dE}$$

where Φ(t,E) is the differential fluence spectrum of the beam after traversing a thickness *t*. If $E{\left(\mu_{\mathrm{en}}/\rho \right)}_{a}$ is independent of energy, then Equation (3) reduces to the total fluence ratio as per JB and $B_{k}\to B_{\mathrm{JB}}$.

Changes in the beam spectrum can be quite large. McDermott^11^ has calculated the spectrum of radiation that emerges from a concrete roof as part of the calculation of linac skyshine. The incident energy spectrum is taken from Sheikh‐Bagheri and Rogers as in the JB paper. For an 18 MV, 40 × 40 cm^2^ (at isocenter) beam and a concrete barrier thickness of 91 cm, the average energy of the incident beam is 4.9 MeV and the average energy of the transmitted beam is 10.5 MeV. For a 4 MV, 40 × 40 cm^2^ beam traversing 61 cm of concrete the average incident energy is 1.5 MeV and the average transmitted energy is 2.7 MeV.

In 2013, K2 published extensive tables of TVL data for concrete, lead, and steel.^6^ These authors used a beam with a half opening angle of 12.7° corresponding in area to a square of side length 40 cm at a distance of 100 cm from the source. The distance to the distal surface of the barrier is 6.0 m and *d*_w_ = 0.3 m. The authors first calculate build‐up factors for monoenergetic beams. The monoenergetic transmission *b*(*t*, *E*) is computed from the build‐up factors and then it is fitted to a three‐parameter model. The linac transmission factors are then calculated by integrating over the linac spectrum as follows:

(4) $$B_{K2}\left(t\right)=\frac{\int_{0}^{\infty }E\Phi \left(t,E\right){\left(\mu_{\mathrm{en}}/\rho \right)}_{a}b\left(t,E\right)dE}{\int_{0}^{\infty }E\Phi \left(0,E\right){\left(\mu_{\mathrm{en}}/\rho \right)}_{a}dE}$$

The beam spectra of Sheikh‐Bagheri and Rogers^9^ have been used. The K2 definition of *B* is thus based on a ratio of air kerma values. This is a somewhat roundabout method of computing the transmission, but the authors point out that this can be applied to compute transmission factors for scatter and leakage as well as primary radiation. In contrast to the NL TVL data, K2 have used more recently calculated beam spectra and an opening angle that corresponds to a 40 × 40 cm^2^ field.

TVL data for concrete from NCRP151, IPEM75, IAEA47, K2, and JB are summarized in Table 1. For the JB data, and for thickness *t* > TVL_1_ + TVL_2_, the barrier transmission is given by:

(5) $$B\left(f,d_{w}\right)=10^{-2}\times 10^{-\left(\frac{t-\mathrm{TV}L_{1}-\mathrm{TV}L_{2}}{\mathrm{TV}L_{3}}\right)},$$

where TVL_1_, TVL_2_, and TVL_3_ depend on *f* (side length of the equivalent square) and *d*_w_.

**TABLE 1:** Tenth value layer (TVL) data for concrete

	NCRP151/IPEM75		Karoui and Kharrati (2013)		IAEA47	Jaradat and Biggs (2007)^a^		
Energy (MV)	TVL_1_ (cm)	TVL_e_ (cm)	TVL_1_ (cm)	TVL_e_ (cm)	TVL (cm)	TVL_1_ (cm)	TVL_2_ (cm)	TVL_3_ (cm)
4	35	30	29.66	27.59	29.0	36.5	26.5	27.5
6	37	33	33.91	33.07	34.3	40.0	31.5	34.5
10	41	37	41.40	40.18	38.9	50.5	39.0	40.0
15	44	41	43.80	42.85	43.2	53.0	43.0	44.0
18	45	43	46.72	45.22	44.5	58.0	44.0	48.0

Abbreviations: IAEA47, IAEA Report No. 47; IPEM75, IPEM Report No. 75; NCRP151, NCRP Report No. 151.

^a^ 12° opening half angle, distal distance from barrier wall is 0.3 m.

All of the data in Table 1 are based on a concrete density of 2.35 g/cm^3^. The values from JB are for a 12° half opening angle and a distal distance from the barrier of *d*_w_ = 0.3 m. The IAEA47 report only gives a single value of the TVL for each energy. The IAEA values are “approximate values based on large attenuation.”^3^ The TVL data in Martin and McGinley^2^ are the same as in NCRP151 with the exception of the TVL_e_ for 10 MV radiation (TVL_e_ = 38.9 cm vs. 37.0 cm in NCRP151) that was taken from the IPEM75 (first edition). According to JB, the average uncertainty in their TVL data, averaged over all values of *d*_w_ for a 14° half opening angle is on the order of 1 cm. It is not clear from any of the references cited above, how the TVL values were derived from barrier transmission data. For example, how were the values of TVL_e_ in NCRP151 obtained from the values of TVL_2_ and TVL_3_ listed in NL?

**FIGURE 1:**
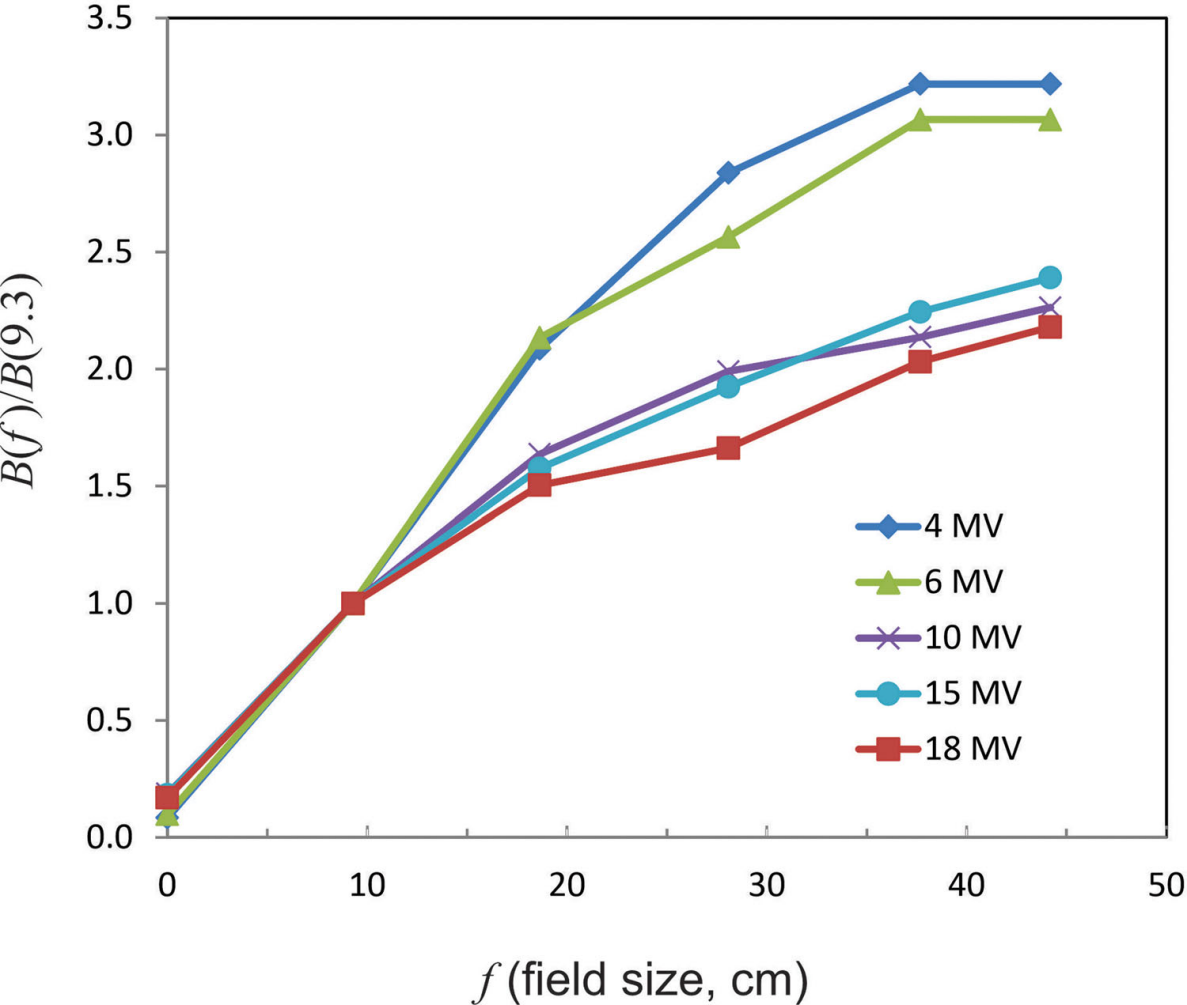
Relative dependence of barrier transmission on field size (length of the side of the equivalent square) for beams with *d*_w_ = 0.3 m based on the Jaradat and Biggs (JB) data for tenth value layer (TVL). Values are normalized to a field size of 9.3 cm (half opening angle of 3°). This is for a concrete thickness of 150 cm. Low‐energy beams show the largest field size dependence. The barrier transmission is up to two to three times larger for the largest field sizes compared to a 9.3 × 9.3 cm^2^ field

As the field size increases, it is expected that the barrier transmission will rise because the beam intercepts more scattering centers. When the field size is sufficiently large, the additional scattered photons will be unable to reach the central axis and it is anticipated that the barrier transmission will saturate. Figure 1 shows the relative field size dependence of the barrier transmission for the JB data (*d*_w_ = 0.3 m) as a function of the equivalent square field size *f* at isocenter for energies of 4–18 MV. Equivalent squares for these circular fields have been computed using the formula: $f=r\sqrt{\pi }$, where *r* is the radius of the circular field with a given opening angle. The transmission values have been normalized to the value for *f* = 9.3 cm (3° half opening angle). The plotted values are for a barrier thickness of 150 cm and for points 0.3 m distal to the surface of the barrier. The results are qualitatively similar for other thicknesses. The 4 MV beam shows the most dependence on field size and the 18 MV beam the least. This is likely due to the fact that lower energy photons have a higher probability of scattering through large angles. The maximum relative value of *B* for 4 and 6 MV is over three times its value for *f* = 9.3 cm. The 4 and 6 MV beams show evidence of saturation at the largest field sizes.

The qualitative nature of the field size dependence is confirmed by measurements. In a paper by Elder et al.,^17^ measured transmission data are reported for a linac roof (primary barrier) based on the ratio of exposure measurements (or equivalently air kerma ratios). The exposure rate was measured at isocenter and at a distance of 0.9 m above the roof top. For 6 MV, the ratio of the barrier transmission for a 40 × 40 cm^2^ field to that for a 10 × 10 cm^2^ field is 2.0 and for 10 MV this ratio is 1.6. The thickness and composition of the roof are unspecified but *B* = 0.052 for 6 MV (40 × 40 cm^2^) and *B* = 0.082 for 10 MV.

The scatter contribution to the transmitted radiation arguably depends on the field size *at the barrier* rather than the opening angle of the beam at the isocenter. The distance used by JB was 6.0 m from the target to the distal side of the barrier. At distances other than 6.0 m the area will be different. For a square field, it is possible to define an effective half opening angle leading to the same area at the barrier as used in the JB calculations, viz:

(6) $$\tan\theta_{e}=\frac{6f}{d_{b}\sqrt{\pi }},$$

where *f* is the side length of the square (in meters) as measured at the isocenter and *d*_b_ is the distance to the distal face of the barrier from the target (in meters). When consulting the tables in JB, use of the effective angle is likely to lead to the most accurate TVL data.

Although Jaradat and Biggs state in their conclusion that “the data at large angles agree with NCRP Report No. 151,” the JB data actually lead to substantially different transmission values, differing by as much as a factor of 4 from NCRP151.

**FIGURE 2:**
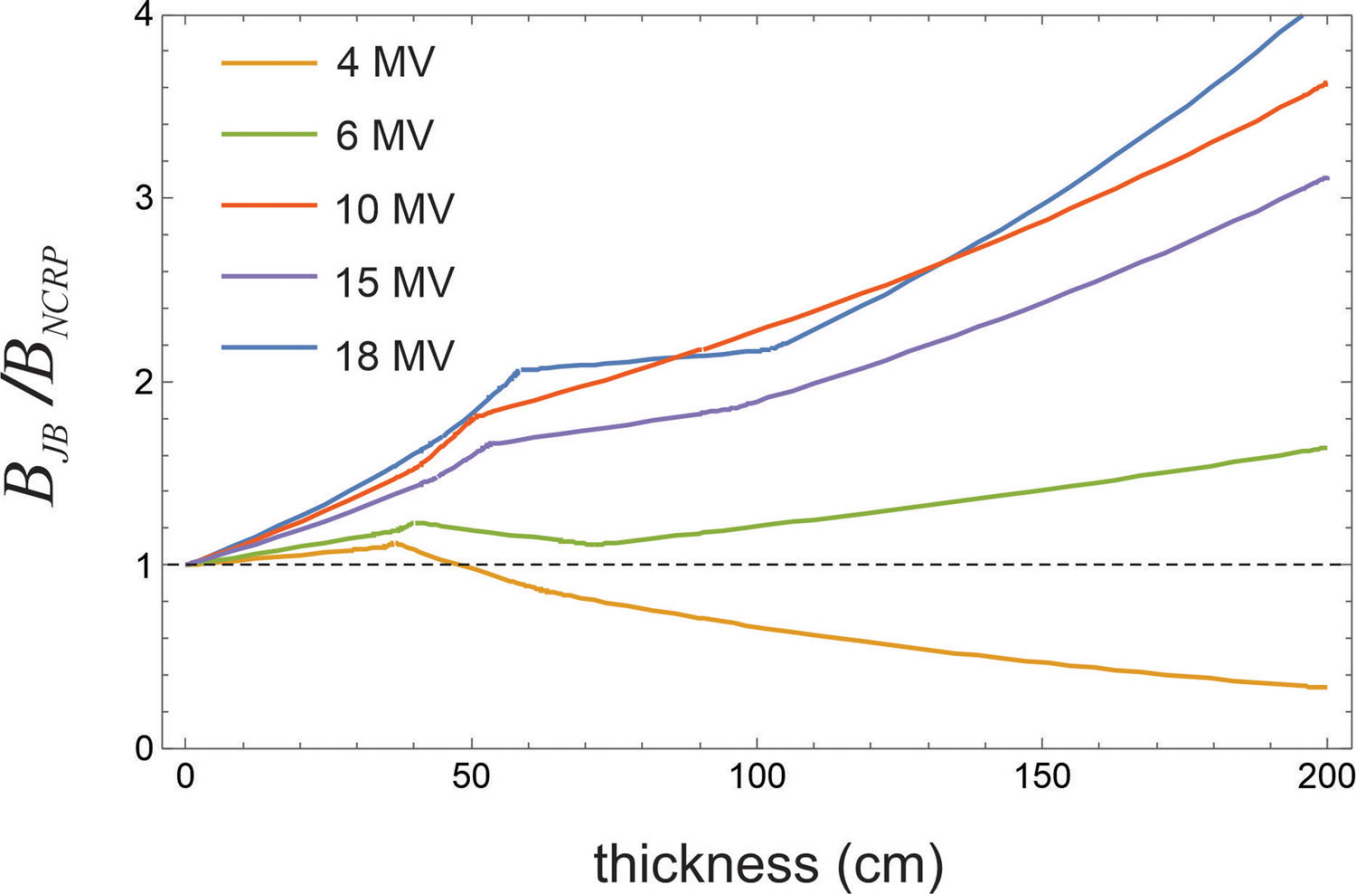
The ratio of the barrier transmission for the Jaradat and Biggs (JB) tenth value layer (TVL) data relative to the NCRP Report No. 151 (NCRP151) data as a function of the thickness of the concrete. The JB data are for half beam opening angle 14° and *d*_w_ = 0.3 m. The JB computed barrier transmission is larger than for NCRP151 for energies of 6 MV and above. The ratio increases with increasing concrete thickness. The ratio is as high as a factor of 4 for 18 MV and concrete thickness of 200 cm. The ratio is less than 1 for 4 MV and can be as small as 0.4 for a concrete thickness of 200 cm

Figure 2 shows the ratio of the barrier transmission calculated from the JB data to that calculated using the NCRP151 data as a function of the thickness of the barrier. This is for a 14° half opening angle and *d*_w_ = 0.3 m. For all beam energies >4 MV, the transmission predicted by JB is greater than that predicted by NCRP151. The *B* ratio is highest for higher energies and thicker barriers. The ratio is as high as a factor of 4 for 18 MV and a thickness of 200 cm. The agreement with NCRP151 is moderately good for 6 MV. For 4 MV the ratio is less than 1 and becomes smaller for thicker barriers. In this case, the ratio is as little as 0.4 for thickness 200 cm. The IAEA47 computed barrier transmission agrees a little better with JB than NCRP151. The ratio of the JB predicted *B* to that for the IAEA data shows similar characteristics to NCRP151 (Figure 2). The ratio is greater than 1.0 for all energies and all thickness with the exception of 4 MV with thickness >150 cm, where it dips slightly below 1.0. The ratio is greater than 3 for 18 MV and thickness greater than 200 cm.

The NL TVL values have only been computed for energies of 6, 10, and 25 MV. The NCRP151 TVL_1_ data for 15 and 18 MV have been derived by linear interpolation between the NL values for 10 and 25 MV. The NCRP151 values of TVL for 4 MV have been derived by a linear *extrapolation* from the NL TVL values for 6 and 10 MV. The 1984 NL beam spectra may not accurately represent contemporary linacs. The average photon energy for the 6 MV NL beam is 1.76 MeV whereas the corresponding value for JB is 1.75 MeV. This may explain the relatively good agreement between JB and NCRP151 for 6 MV. For 10 MV however, the NL average photon energy is 2.6 MeV whereas the more recent spectra used by JB (based on Sheikh‐Bagheri and Rogers) has an average energy of 3.3 MeV. The linear attenuation coefficients in concrete are 9.3 m^−1^ for 2.6 MeV and 8.25 m^−1^ for 3.3 MeV. The ratio of narrow beam monoenergetic transmission for these two energies traversing 1.5 m of concrete is 4.8. This suggests an explanation for the discrepancies between the NL and JB TVL data illustrated in Figure 2. Furthermore, if the NL TVL values are too small for 10 MV, extrapolation to 4 MV will result in TVL values that are too large, consistent with the ratios shown in Figure 2 for 4 MV.

The fact that *B* depends on *d*_w_ means that radiation levels will not follow the inverse square law beyond the distal surface of the barrier. This is not surprising because the wall acts like a source of radiation. This pseudo‐source does not act like a point source until the distance from the source *d* >> *d*_b_(*f* + 1), where *f* is the side length of the equivalent square at the isocenter in units of meters. This lack of inverse square behavior has been reported previously by McGinley^12^ and Biggs^13^. The distance dependence is given by *B*(*d*_w_)/*d* ^2^. The most accurate predictions must account for the value of *d*_w_. The JB TVL data show that there is a more rapid fall off with distance than would be predicted using *B*(*d*_w_ = 0.3 m) alone and therefore the use of *d*_w_ = 0.3 m (as compared to *d*_w_ = 1 or 2 m) will lead to conservative estimates of the radiation levels for *d*_w_ > 0.3 m. As an example, for 18 MV and concrete thickness 150 cm *B*(*d*_w_ = 2 m)/*B*(*d*_w_ = 0.3) is only 0.60. Therefore, at a distance of 2.0 m from the wall, the IDR is only 60% of the expected value based on *d*_w_ = 0.3 m.

It is instructive to repeat one of the example problems discussed in NCRP151 (page 120, primary barrier at location D), using the JB and K2 TVL data. This is the control room of the linac and therefore it is a controlled area. The parameters are *W*(18 MV) = 450 Gy/week, *U* = 0.25, *T* = 1, *d* = 7.2 m, and permissible radiation level *P* = 10 mrem/week.^4^ The necessary value of the barrier transmission is *B* = 4.61 × 10^−5^ . Using the TVL data of NCRP151, the concrete thickness needs to be 189 cm. Using the JB data for this thickness gives *B* = 1.75 × 10^−4^. Based on this, the radiation level at point D, is expected to be 38 mrem/week. This is almost a factor of 4 above the recommended limit. The thickness of the concrete necessary using the JB data is about 220 cm. If the JB data are accurate, the use of NCRP151 data may lead to the expense and trouble of remediation unless it is argued that the use of such large field sizes is rare. This is not however, an argument that has been made in NCRP151. The conservative approach is to assume the worst case scenario and to require the shielding to meet regulatory (or ALARA) limits under large field conditions. If one uses the IAEA47 data (*B* = 5.66 × 10^−5^) the predicted value of *P* = 12 mrem/week, low by about a factor of 3 compared to JB. For the K2 TVL, *B* = 7.14 × 10^−5^ and the weekly radiation level is 16 mrem/week.

### 3.2 Instantaneous air kerma measurements

Unambiguous published IDR measurements for primary barriers seem hard to come by. A master's thesis by Kildea quotes measured radiation readings outside primary concrete barriers for 6 and 18 MV.^14^ For two barriers at 6 MV, the quoted ratios: (measured)/(NCRP151 predicted), are 2.1 and 2.5, and for one 18 MV barrier this ratio is 2.7. For a different 18 MV barrier however, they report a ratio of 0.13. The barrier thicknesses are not reported.

Rijken et al.^15^ report 10 MV IDR measurements for 27 primary concrete barriers with thicknesses ranging from 120 to 250 cm. The field size at isocenter was 40 × 40 cm^2^. The measurements were made with “large volume ion chamber survey meters.” All of the measurements are normalized to a distance of 6 m from the isocenter. The measured “dose” rates have been compared to calculated dose rates using TVL data from NCRP151, IAEA47, and IPEM75 (first edition). The calculated dose rate is given by:

(7) $$\dot{D}=B\frac{\dot{D}\left(0\right)}{{\left(1+r\right)}^{2}},$$

where *B* is the barrier transmission based on the published TVL values, $\dot{D}\left(0\right)$ is the dose rate at the isocenter, which was taken as 6.6 Gy/min, and *r* is the distance from the *isocenter* to the point of interest.

**FIGURE 3:**
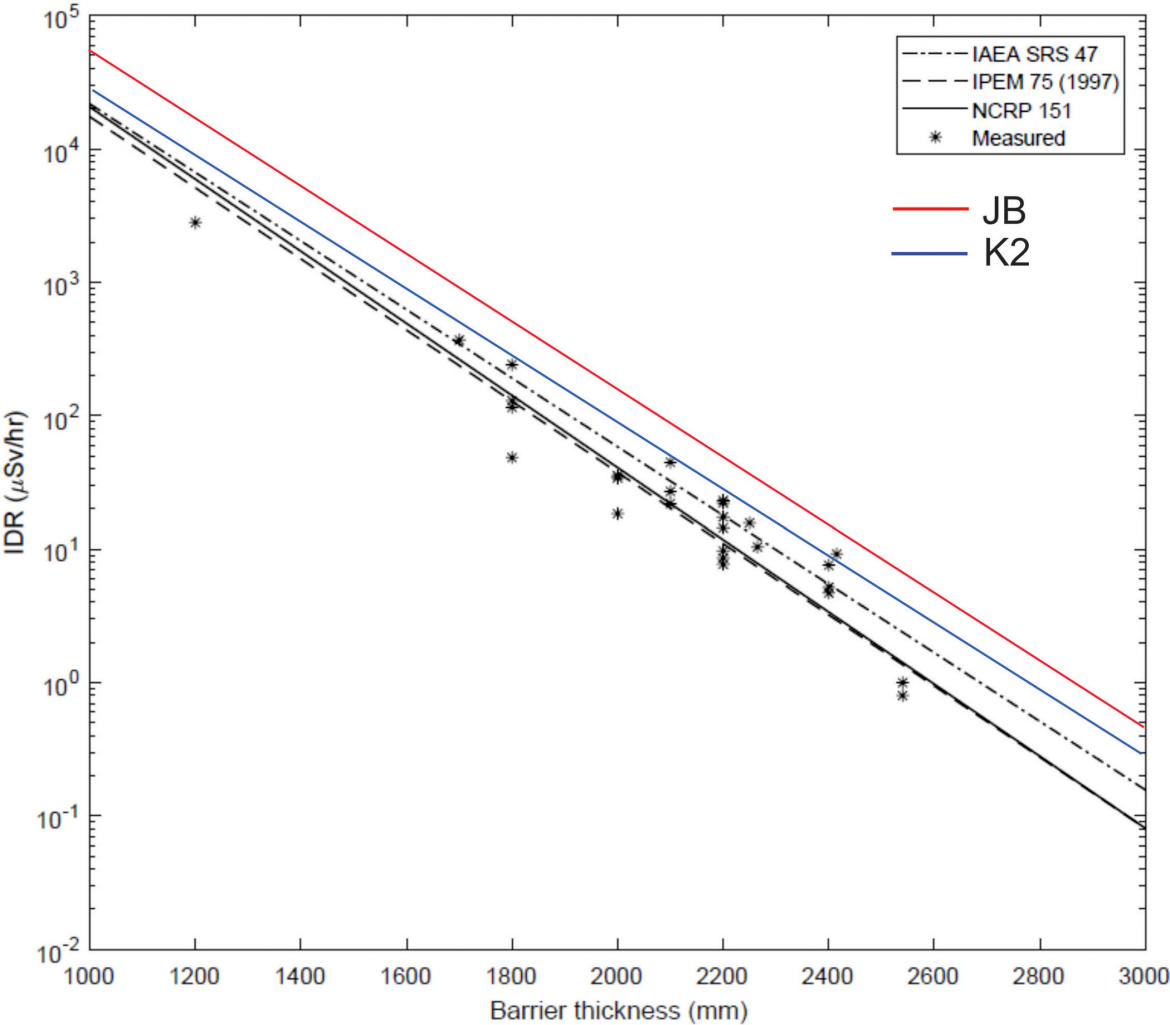
A modified version of Figure 3 from Rijken et al. showing a graph of the log of the 10 MV measured instantaneous dose rates (IDR) (in μSv/h) versus concrete thickness (in mm) for 27 barriers. The field size is 40 × 40 cm^2^ and all measured data have been normalized to a distance from the isocenter of 6 m and a dose rate at isocenter of 6.6 Gy/min. The predicted values of the IDR are also shown. The solid curve is for NCRP Report No. 151 (NCRP151), the dashed curve for IPEM Report No. 75 (IPEM75), and the dot dashed curve is for IAEA Report No. 47 (IAEA47). The red curve for the Jaradat and Biggs (JB) data (12° half opening angle, *d*_w_ = 0.3 m) and the blue curve for the Karoui and Kharrati (K2) data have been added to the graph. There is a large spread in the IDR for different barriers of the same thickness. Although the NCRP prediction curve goes through the center of the measured values, it is clear that in many cases it significantly underestimates the measured IDR. The JB tenth value layer (TVL) data always overestimate the measured IDR

Figure 3 is a modified version of Rijken's Figure 3. This shows a graph of the log of the IDR versus the barrier thickness for the 27 measurements along with the predictions based on TVL data from NCRP151, IAEA47, IPEM75, JB, and K2. The IPEM data appear to be from the first edition of this reference; the second edition uses data from NCRP151. The distance from the isocenter to the point of measurement has been normalized to 6.0 m and the dose rate at isocenter is a standard 6.6 Gy/min. There is a large spread in the measured IDR for many of the barriers having the same thickness. For example, for concrete thickness 180 cm, the lowest measured value is 50 μSv/h and the highest is 250 μSv/h. Thus there is a difference of a factor of 5 in measured IDR for barriers having the same thickness! Rijken et al. do not comment on this rather large spread in values. Although the NCRP151 prediction curve goes through the center of the scattered measured data points, it is clear that it sometimes underestimates the IDR by large factors. For example, for a thickness of 240 cm, the NCRP151 prediction is low by a factor of 3. The IDR predicted by the JB data are always higher than the measured values in every case (at least for these 10 MV data).

Table 2 lists measured and calculated IDRs for the primary walls and roof of the Lenox facility for beam energies of 6, 10, and 15 MV and for the Royal Oak facility for energies of 6 and 18 MV. Measurements were made on several occasions with multiple calibrated ionization chamber survey meters (Fluke model 451P) at a distance of 0.3 m from side walls and at a distance of 1.0 m from the roof top (on the central axis). For Lenox measurements the field size was 40 × 40 cm^2^ and for Royal Oak it was 34 × 34 cm^2^. The collimator angle was 45° in all cases. The rep rates in MU/min were recorded at the console. The JB TVL data for a 9° half opening angle have been used.

**TABLE 2:** Comparison between measured and predicted instantaneous dose rates (IDR)

Barrier	Thickness (cm)	Distance (m)^a^	Energy	Rep rate (MU/min)	NCRP151 (mrem/h)	JB (mrem/h)	K2	Measured (mrem/h)	Measured/NCRP151	Measured/JB	Measured/K2
Northwest (interior wall), Lenox^b^	183	8.3	15	706	33	67	45	58	1.8	0.86	1.3
			10	585	9.9	29	20	22	2.2	0.75	1.1
			6	572	2.6	3.2	2.1	5.3	2.0	1.6	2.5
Northeast^c^ (exterior wall), Lenox^b^	183	8.0	15	706	23	48	32	38.5	1.7	0.80	1.2
			10	585	6.8	20	14	16	2.4	0.80	1.2
			6	572	1.7	2.1	1.4	3.3	2.0	1.6	2.4
Roof, Lenox^b^	168	6.0	15	706	141	283^d^	187	155^d^s	1.1	0.55	0.83
			10	585	47	135	88	67	1.4	0.49	0.76
			6	572	14	15	11	15.2	1.1	1.0	1.3
SL2^e^ (Royal Oak) (34 × 34 cm^2^)	216	6.6	18	499	5.9	17	10	8.5	1.5	0.51	0.85
			6	485	0.20	0.27	0.17	0.30	1.5	1.1	1.9

Abbreviations: JB, Jaradat and Biggs; K2, Karoui and Kharrati; NCRP151, NCRP Report No. 151.

^a^ ^”^Distance” is the distance from the target to a point 0.3 m beyond the distal surface of the barrier, except for roof.

^b^ Lenox corrected for measured concrete density.

^c^ For northeast wall a correction factor for the brick façade has been included.

^d^ For *d*_w_ = 1.0 m.

^e^ SL2 there is a steel laser mounting plate approximately 1 cm thick: *B* ∼ 0.8 for both 18 and 6 MV.

Confounding factors have been accounted for wherever they are known. The TVL for Lenox were scaled by the ratio of standard concrete density to the certified construction density (1.026). In the outside wall (northeast) there is a brick facade. A rough estimate of the transmission of the brick is estimated by assuming the brick is 10 cm thick and has a density of 1.9 g/cm^3^. The equivalent concrete thickness is roughly 8 cm. The transmission at 15 MV for this much concrete is *B* ∼ 0.66. On the Lenox roof there is additional building material of an unknown nature on top of the concrete. For this reason, the measured values are expected to be somewhat lower than predicted. For the Royal Oak facility, the transmission of a 1 cm thick steel laser mounting plate has been included.

The predicted values of the instantaneous air kerma rate are computed from the formula: *P* = *BW*/*d* ^2^, where *d* is the distance from the target to a point 0.3 m beyond the distal surface of the concrete. The instantaneous value of the workload *W* is assumed to be equal to the dose rate of the linac for a 10 × 10 cm^2^ field at a depth of *d*_max_ in a large water phantom. The linacs in Table 2 are calibrated so that 1 MU = 1.00 cGy for a 10 × 10 cm^2^ field at a distance of 100 cm + *d*_max_ and at depth *d*_max_ on the central axis.

All of the measured IDR values are higher than predicted using NCRP151 TVL data. The ratio of measured to predicted IDR is as high as 2.5. For the JB TVL data, the measured values are lower for 18, 15, and 10 MV, but higher for 6 MV. For the K2 TVL, most of the ratios are $\underset{∼}{>}1.0$ except for 6 MV where the measured values are about a factor of 2 higher than predicted.

## 4 DISCUSSION

There are many possible confounding factors in the assessment of the barrier transmission of a concrete wall or ceiling. The first of these is the density and composition of the concrete. According to Walker and Grotenhuis,^16^ quoted in NCRP151, the density of “ordinary concrete” can range from *ρ* = 2.09 to 2.50 g/cm^3^. If we assume that the composition is approximately constant, then the TVL should be scaled by the ratio of the standard density to the actual density. Let us denote *B*ʹ as the transmission for non‐standard density, then $\log\left(B^{\prime }/B\right)=t\left(1-\rho /\rho_{0}\right)/\mathrm{TV}L_{3}$, where *t* is the thickness, *ρ*_0_ is 2.35 g/cm^3^, and *B* is the standard transmission. For 4 MV and a thickness of 150 cm, *B*ʹ/*B* ranges from 0.45 to 4.0. For 18 MV and a thickness of 200 cm, *B*ʹ/*B* ranges from 0.55 to 2.8. The measurements reported for all beam energies in Table 2 for the Lenox vault would be in agreement (within about 20%) with the predictions of NCRP151 if the concrete density is actually 2.16 g/cm^3^. This density is at the lower end of the range specified above and is contradicted by the construction firm's certification value of 2.29 g/cm^3^, but it cannot be totally ruled out.

Rarely, if ever, is concrete the only material in a primary barrier. The concrete itself is embedded with steel rebar. As discussed in NCRP151, the presence of rebar is expected to lead to decreased transmission. In addition, there may be other materials in front of, or behind the barrier (masonry, laser mounting plates, dry wall, etc.). As shown in Figure 1, the transmission can be very sensitive to the field size *at the barrier*, an effect not generally accounted for. Furthermore, the distance from the distal face of the barrier to the point of measurement can have a significant effect. All of these factors are likely responsible for the large dispersion in the measured IDR.

Extra material in or on the barrier will lead to a *lower* measured IDR than predicted. This is an acceptable circumstance and consistent with the ALARA philosophy. Despite this, the measured IDR are often *higher* than the predictions based on the TVL data derived from NL. The TVL values for concrete in NCRP151, IAEA47, and IPEM75 can lead to underestimates of the IDR by up to a factor 3 or 4. This could result in the need for expensive and time‐consuming remediation.

It is difficult to conclude for certain which TVL data set is most accurate. It is clear however, that the JB data for 14° half opening angle almost always predict higher than measured IDR for 10, 15, and 18 MV. For this reason, the most conservative approach would be to adopt the JB data until more accurate data become available. For simplicity, and in the spirit of ALARA, we recommend that the TVL*_n_* always be taken for the 14° half opening angle (with *d*_w_ = 0.3 m). We recommend using the data for *d*_w_ = 0.3 for the roof also, even though the relevant distance is arguably 1.0 m (to individual's waist or trunk)—again for simplicity. This will overestimate the IDR at 1 m from the top of the concrete.

It is to be noted that for primary barriers, Martin and McGinley^2^ suggest multiplication of the calculated dose rate by a “recommended dose rate margin.” These authors suggest a factor of 2–3 for concrete that is cast in place. This is a rather large correction factor. No such correction factor is recommended by NCRP151. The need for this factor could be due to density variations or it could signal inaccurate TVL data.

For contiguous areas it is important that survey measurements be made at a distance of 0.3 m from the primary barrier as *B* depends fairly sensitively on *d*_w_. A 40 × 40 cm^2^ field size should be used for the survey. On page 100 of NCRP151 it says: “The primary barriers are surveyed utilizing the maximum field size without a phantom in the beam.” This is the worst case scenario as such field sizes are rarely, if ever, used to treat patients. As shown in Figure 1, smaller field sizes will have a considerably smaller transmission. A 10 × 10 cm^2^ field size may have a transmission of about a factor of 3 lower than 40 × 40 cm^2^.

The values of the barrier transmission *B*, from which the TVL are derived, depend on the definition of *B*. The NL, JB, and K2 TVL data are based on implicit definitions that are not strictly correct. The definition of *B* should be mathematically consistent with its use in the equation *P* = *BWUT*/*d* ^2^. According to NCRP151: “*W* = workload or photon absorbed dose delivered at 1 m from the X‐ray target per week (Gy week^−1^).” The values of the TVL derived from NL are not consistent with this equation. Neither are the values computed by JB or K2. For NL and K2, *B* is a ratio of air kerma and in the JB case it is a ratio of total fluence. However, the dose rate at the isocenter in a large water phantom at depth *d*_max_ will be approximately equal to the air kerma at the isocenter.

We submit that the proper definition of the barrier transmission is:

(8) $$B=\frac{{\dot{K}}_{a}/1\;m^{2}}{{\dot{D}}_{0}/d^{2}},$$

where ${\dot{K}}_{a}$ is the instantaneous air kerma rate measured in Sv/h (= Gy/h) at the point of interest (0.3 m beyond the distal face of the barrier and on the central axis) for a 40 × 40 cm^2^ field (as measured at isocenter), and *d* is the distance from the source in meters. We presume that this is what an ideal ionization chamber survey meter measures. The cumulative value of this over some interval of time is given by the numerator of Equation (3). ${\dot{D}}_{0}$ is the instantaneous absorbed dose rate (workload in Gy/h) in a large water phantom on the central axis at a depth of *d*_max_ and at a distance of 1.0 m from the source for a 10 × 10 cm^2^ field size. If the beam calibration is at a point that is 1.0 m from the source at *d*_max_ for a 10 × 10 cm^2^ field and if 1 cGy = 1 MU at the calibration point then ${\dot{D}}_{0}=\dot{\mu }\times 0.01\;\;(\mathrm{Gy}/\mathrm{MU})$, where $\dot{\mu }$ is the instantaneous rep rate (in MU/h). The rep rate in MU/min can be read directly from the linac console. The definition of *B* given by Equation (8) is consistent with the use of this factor in the equation *P* = *BWUT*/*d* ^2^ and can be tied directly to the rep rate of the linac as read at the console.

## 5 CONCLUSIONS

Predicted values of the air kerma rates outside concrete primary linac barriers vary considerably (up to a factor of 3 or 4) depending on the source of the TVL data. The TVL data quoted by NCRP151, IPEM75, and IAEA47 are all derived from a 1984 paper by NL. The NCRP151 TVL data for 15 and 18 MV appear to be based on interpolations between 10 and 25 MV. The NCRP151 4 MV TVL data are based on an extrapolation from the data at 6 and 10 MV. The differences between the NL TVL data and the more recently calculated TVL data may be due to differences in the beam spectra, particularly for 10 MV.

The use of NCRP151 TVL data leads to predictions of the instantaneous air kerma rate that are up to a factor of 3 *lower* than measured values, potentially requiring expensive remediation. The JB values of the TVL generally overestimate the measured instantaneous air kerma rates and therefore the use of these values will provide very conservative estimates of concrete primary barrier transmission.

It would be helpful to have published reference TVL data based on barrier transmission values calculated directly, using a Monte Carlo algorithm along with the definition given in Equation (8). This would provide mathematical consistency with its use as a multiplier of the workload as defined in NCRP151. The calculated air kerma should explicitly account for the large changes in the beam spectrum that occur as the beam traverses the barrier. These computations should be parametrized by the field size *at* the barrier and the distal distance from the barrier to the point of interest. This should be done for energies of 4, 6, 10, 15, and 18 MV without energy interpolation or extrapolation.

## CONFLICT OF INTEREST

The authors declare that there is no conflict of interest.

## AUTHOR CONTRIBUTIONS

Patrick N. McDermott performed original shielding design for Lenox facility, conducted a radiation survey, and did the writing for this paper. Michael D. Sigler participated in the Lenox radiation survey and checked some construction details. Ian P. Lake checked the barrier calculations and participated in the survey. Danielle Lack checked the barrier calculations and communicated with the construction firm.

## Notes

1 The term IDR is used throughout. This is usually measured with an ionization chamber survey meter and therefore it is not really the absorbed dose but rather the instantaneous air kerma rate.^ia^ With the exception of the NCRP151 TVL for 30 MV.

2 The IAEA47 TVL data are identical to data listed in Tables 2–5 of the Varian “Designers’ Desk Reference,” Vol. 16, No. 4 (2016). No reference is given for these data.

3 A horizontal linac beam with an opening half angle of 14° begins to impinge on the floor at a distance of 5.2 m from the source, assuming an isocenter height of 1.3 m above floor level.

4 There is a misprint in NCRP151: they say TVL_1_ = 47 cm in the example but it should be 45 cm according to Table B.2.
